# Scientific issues related to the cytology proficiency testing regulations

**DOI:** 10.1186/1742-6413-3-11

**Published:** 2006-04-18

**Authors:** George Birdsong, Lydia Howell, Karen Atkison, R Marshall Austin, Marluce Bibbo, Thomas A Bonfiglio, Diane D Davey, Catherine Keebler, Dina Mody, Lynnette Savaloja, Jacalyn Papillo, Marianne Prey, Stephen Raab, Brenda L Schultz, Diane Solomon

**Affiliations:** 1 Grady Health System, 80 Jessie Hill Junior Drive, Atlanta, GA 30303, USA; 2 University of California Davis Medical Center, Office of Academic Affairs, PSSB, Suite 2500, 4150 V Street, Sacramento, CA 95817, USA; 3 TriPath Imaging, Inc., 780 Plantation Drive, Burlington, NC 27215, USA; 4 University of Pittsburgh, Department of Pathology, 300 Halket Street, Pittsburgh, PA 15213, USA; 5 Thomas Jefferson University Hospital, Cytology Lab, 132 S. 10^th ^Street, RM 260 Main, Philadelphia, PA 19107-5244, USA; 6 University of Rochester Medical Center, Cytopathology Unit, 601 Elmwood Ave., Box 626, Rochester, NY, USA; 7 University of Kentucky Medical Center, Pathology & Laboratory Medicine, 800 Rose Street, MS 117, Lexington, KY 40536, USA; 8 International Academy of Cytology, Chicago, IL 60615, USA; 9 The Methodist Hospital, Department of Pathology, 6565 Fannin Street, MS205, Houston, TX 77030, USA; 10 Regions Hospital, Pathology Department, 640 Jackson Street, Saint Paul, MN 55076, USA; 11 Quest Diagnostics Incorporated, Department of Pathology, 2040 Concourse Drive, St. Louis, MO 63146, USA; 12 UPMC Health System, UPMC-Cancer Pavilion, 5150 Centre Avenue, Room 306, Pittsburgh, PA 15232, USA; 13 ASCT Services, 1500 Sunday Drive, Suite 102, Raleigh, NC 27607, USA; 14 Consultant, Bethesda, MD 20817, USA

## Abstract

The member organizations of the Cytology Education and Technology Consortium believe there are significant flaws in current cytology proficiency testing regulations. The most immediate needed modifications include lengthening the required testing interval, utilizing stringently validated and continuously monitored slides, changing the grading scheme, and changing the focus of the test from the individual to laboratory level testing. Integration of new computer-assisted and located-guided screening technologies into the testing protocols is necessary for the testing protocol to be compliant with the law.

## Preamble

In the following document, the Cytopathology Education and Technology Consortium (CETC) states in detail its concerns with technical and scientific aspects of the federal cytology proficiency testing (PT) criteria established in 1992 regulations implementing the *Clinical Laboratory Improvement Amendments of 1988 (CLIA)*. The CETC continues to be concerned that the program has fundamental flaws and therefore supports the larger pathology community in urging HHS to suspend and halt further implementation of the program until a thorough re-evaluation of its approach, relevancy and validity can be conducted. The procedures for evaluation of the quality of laboratory testing should be developed in conjunction with knowledgeable professional organizations; they should not be relegated solely to implementation of the examination. Also, certification examinations administered by pertinent medical specialty boards and allied health credentialing agencies should be taken into account in determining whether or not competence has been adequately demonstrated. Our views, however, are not limited to those described in this communication. Some members of the consortium believe strongly that adjusting the current regulation will not by itself correct this flawed program and that an alternative approach must be developed that may require changes to the underlying statute as well as changes to the regulation. The CETC urges CMS to consider all necessary changes whether they be regulatory or statutory in order to revise this program. The CETC will be reviewing all the pertinent regulations [1,2] and responding with detailed comments, which will include justification and the impact of our suggested changes.

Proficiency testing (PT) in gynecologic cytology has been a controversial topic for many years. Though mandated by the federal government seventeen years ago as part of the Clinical Laboratory Improvement Amendment (CLIA '88), it had been implemented in only one state prior to 2005. Challenges preventing widespread implementation have included the inability to replicate normal working conditions, the subjective nature of cytologic interpretation, absence of a "gold standard" against which test results can be compared, and confidence that test performance adequately correlates with proficiency and competency of the practicing professional and improves patient care. In addition, PT has been directed chiefly at the level of the individual cytotechnologist and cytopathologist, and has not addressed performance of the entire laboratory or aspects of the Pap test process other than microscopic evaluation.

In the fall of 2004, the Center for Medicare Services (CMS) announced its approval of a proficiency test developed by the Midwest Institute for Medical Education (MIME), and its planned implementation in 2005 to fulfill the CLIA mandate. Members of the Cytology Education and Technology Consortium (CETC), an organization composed of representatives of the American Society of Cytopathology, American Society for Clinical Pathology, the American Society for Cytotechnology, the College of American Pathologists, the International Academy of Cytology and the Papanicolaou Society of Cytopathology, met on November 15, 2004 to discuss CMS's announcement. The following are the science-based concerns identified by CETC:

• The frequency of testing is excessive

• Validation of the test slides is inadequate since it is based on the review of only three pathologists. Inadequate validation of test slides could lead to indiscriminate failure of qualified, competent personnel.

• The scoring system and reporting terminology is believed to be inappropriate and unfair. Though these may reflect the current terminology used in Pap test reporting, it does not reflect the clinical implications associated with this terminology in modern practice, particularly regarding recommended follow-up.

• The test does not consider common and important aspects of modern gynecologic cytology practice such as computer-assisted screening or location-guided screening.

• Testing is directed at the level of individuals instead of the level of the laboratory as in all other proficiency testing.

## Discussion

### Testing interval

The CETC recommends that the PT interval be lengthened to five years for most cytology practitioners, rather than the current one year test interval. There is no evidence to suggest that cytology screening and interpretive abilities deteriorate after a year. Cytology assessment is not at all analogous to clinical laboratory PT. Clinical laboratory testing results are very dependent on instrument calibration and reagents, which may vary significantly from lot to lot, necessitating more frequent PT. Less frequent assessment is appropriate for the well-trained cytology professional who is assessing cervical cytology slides on a regular basis.

Certification organizations do not require annual testing to maintain a valid certificate. The 24 medical boards under the American Board of Medical Specialties implemented the Maintenance of Certification initiative a few years ago, and the recertification cycles for these boards range from 6 to 10 years [3]. Test results do not show deterioration during the ten (10) year period (personal communication, M. Lunz). In between the formal examination, board-certified physicians with time-limited certificates are required to show evidence of continuing education and performance improvement initiatives. Cytology laboratories are already subject to many other quality assurance and improvement requirements under CLIA '88, which address daily quality screening practices. The CLIA '88 legislation does not mandate a specific testing interval, stating that such assessment should be "periodic." As long as there are stipulations that individuals new to practice be assessed within a certain time interval, a five-year interval for the great majority of competent practitioners would satisfy the intent of the law.

### Validation of slides

There is concern that the slides used in the MIME test are not well validated. Validated slides are important for meaningful PT. Despite the extensive training undergone by all cytologists, significant interobserver variation in the interpretation of gynecologic cytology specimens is well documented in numerous studies over the past two decades. [4-7] Even experienced cytologists often show significant disagreement in their interpretations of some cases. [8] This interobserver variation may affect the outcome of (PT) in a manner unrelated to actual proficiency of examining slides. For example, a study by Valente and Schantz [9] examined the reproducibility of PT in a workshop setting. One slide with a reference interpretation of low-grade squamous intraepithelial lesion (LSIL) was given that interpretation by examinees 66.7% of the time, whereas two other slides with the same reference interpretation were given the that interpretation by examinees 92% and 94% of the time. This variation may be thought of as representing differing levels of "difficulty" of the cases. In order to be fair and valid, the slides and slide sets presented to different individuals in a proficiency test must be of equal difficulty. Section Sec. 493.945 of the CLIA law specifically states:

"...test sets should be comparable so that equitable testing is achieved within and between proficiency testing providers."

If test slides are not of equivalent difficulty, individual competency assessment is unreliable or inconsistent. [10] While use of a small number of experienced pathologists to assign a reference interpretation for slides used in a PT program is an appropriate part of the overall design of such a program, it should not be the only criterion for selection since interobserver variability amongst examinees can still be quite significant. Once a preliminary reference interpretation is assigned, the difficulty of each slide which will ultimately be included in a PT program must be established by pilot testing, also known as field validation. Field validation consists of statistical assessment of the performance of each slide under actual testing conditions. [11] As a practical consideration for a short examination, all slides in each category should be of the same difficulty, i.e. if there are two HSIL slides on an examination, they should both meet the same validation criteria. The examination should not have one slide which field validated at 75% of responses concordant to the reference interpretation, and another of the same reference interpretation, which validated at 90% concordance.

Slides used for PT should demonstrate that they perform well (i.e., that they can be interpreted in a consistent manner by a significant majority of practicing cytologists) in pilot testing prior to inclusion in proficiency tests. Slides that perform poorly may increase the margin of error of the exam and adversely affect the precision of the pass-fail decision made about candidates. [11] Use of unvalidated slides decreases the likelihood of accurately detecting individuals needing remediation, and increases the likelihood of inconsistent and/or erroneous test outcomes, which could lead to competent cytologists being penalized.

Validation criteria must be stringent in order to minimize the likelihood of spurious results. This is particularly important with regard to HSIL slides since examinees will fail the test if a single HSIL slide is missed. An example of validation criteria used in an interlaboratory comparison program recently published in the peer-reviewed literature by Renshaw [12] includes the following parameters:

1. There must at least 20 responses for each slide, to insure a sufficiently large dataset on which to compute validation statistics.

2. Participants must respond in the correct series at least 90% of the time. (There were three "series" in this study a) unsatisfactory, b) normal, infections, and reparative conditions, and c) epithelial abnormalities and carcinoma.)

3. The standard error of this percentage must be 0.05 or less.

Other criteria included specified rates of concordance to the exact reference interpretation for the LSIL category. The impact of the field validation process on the selection of slides in the program is of interest. 31.8 percent of conventional smears and 15.8 percent of ThinPrep slides with a reference interpretation of LSIL, 9 percent of conventional smears and 17.6 percent of ThinPrep slides with a reference interpretation of high-grade squamous intraepithelial lesion (HSIL) failed to achieve the program's criteria for field validation. In addition, over 50 percent of the slides of either type with a reference interpretation of unsatisfactory failed to achieve the program's criteria for field validation. The reference interpretations for all slides in that study were first agreed upon by consensus of three unmasked experienced cytopathologists as well as the donor laboratory. In addition, slides with an interpretation of any SIL had histologic confirmation.

Another recent study by Renshaw has demonstrated that the robustness of field validation criteria vary with different reference interpretations. [13] The validation criteria for herpes, trichomonas, squamous cell carcinoma, and adenocarcinoma were significantly more robust than the criteria for the interpretations of NILM-NOS, LSIL, and HSIL in that study. The robustness measurement is also a surrogate marker for the ease of slide interpretation; in other words, some reference interpretations are more easily arrived at than others. Differences in difficulty between different reference interpretations must be taken into account in the design of a proficiency test that is fair to all participants. Examination sets should have a similar mix of cases from the high and low robustness groups to avoid having a wide variation in the overall difficulty of the test. In conclusion, the inherent, well demonstrated interobserver variability in the interpretation of Pap tests *must *be taken into consideration in the design of a fair and valid test. Field validation of the slides prior to their use in graded test sets is mandatory for the test to be considered acceptable to the CETC.

A related and important issue is that the validation status of slides in a PT program must be continuously monitored. Slides may become scratched or broken, cover slips may partially detach, and stains may fade. The result of these changes is that the performance of slides may deteriorate from acceptable to unacceptable over time. Slides whose performance falls below the stated validation criteria of the program should be removed from the program and replaced with slides which have been field validated. In addition, provision should be made in the regulations for individuals who fail a test if the slide for any missed question falls below the validation criteria during that round of testing. Individuals in this situation should not be penalized, and if retesting is deemed necessary, there should be no additional cost to the affected individual or his or her institution.

### Proposed grading scheme

The CETC recommends changing the current grading scheme. The grading scheme proposed under the rules published in 1992 (Tables 1, 2) is based on a triage algorithm in use at the time that had been in place since the late sixties. [14] However, with the Bethesda 2001(TBS 2001) Workshop on terminology, [15,16] and the subsequent American Society for Colposcopy and Cervical Pathology (ASCCP) consensus conference on management of patients with Pap test abnormalities reported using TBS 2001, the triage and management guidelines have changed. [17] Under the old guidelines, patients with low-grade lesions (LSIL/HPV/CIN I/mild dysplasia) were often followed by repeat cervical cytology, whereas those with high-grade lesions (HSIL/CIN II and above) were triaged for colposcopy and biopsy. The current management guidelines are evidence-based as a result of our better understanding of Human Papilloma Virus (HPV) biology and the ASCUS Low-Grade Triage Study (ALTS). [18-26] Data from the ALTS trial clearly demonstrated that HPV positive ASC-US and LSIL carry about a 25% risk of harboring a high-grade lesion and hence are referred for immediate colposcopy and biopsy. The subsequent management depends primarily upon the findings from that procedure although if the colposcopic and initial histologic findings are negative, management following a Pap test interpreted as LSIL is more conservative than it is with one interpreted as HSIL.

**Table 1:** Point values (current)

	**Pathologist (Technical Supervisor) 10 Slide Test**			
**Correct Response**	**Examinee Response**			

	A -UNSAT	B -NEGATIVE	C -LSIL	D-HSIL
A -Unsat	10	0	0	0
B -Negative	5	10	0	0
C -LSIL	5	0	10	5
D -HSIL	0	-5	5	10

**Table 2:** Point values (current)

	**Cytotechnologist 10 Slide Test**			
**Correct Response**	Examinee Response			

	A -UNSAT	B -NEGATIVE	C -LSIL	D-HSIL
A -Unsat	10	0	5	5
B -Negative	5	10	5	5
C -LSIL	5	0	10	10
D -HSIL	0	-5	10	10

The College of American Pathologists (CAP) PAP program has been in existence since 1989. The CAP PAP data have demonstrated that it is very difficult to find slides that achieve unanimous consensus as LSIL (low-grade lesions). [4] Even when three experts on the CAP Cytopathology Resource Committee agree with a biopsy-proven case of a LSIL, the slide does not reach field validation from participants approximately 20% of the time. [12] The ALTS trial similarly demonstrated that only 69% of original LSIL interpretations by clinical centers were upheld by the Pathology QC reviewers. [25] LSIL and HSIL are reported as distinct interpretations in TBS 2001, and populations of patients in these two categories do show different follow-up profiles. However, it is recognized that separating these squamous abnormalities in individual cases is not an exact science. Therefore, colposcopy is recommended for both LSIL and HSIL. Hence, the grading scheme penalizing pathologists 5 points for not distinguishing LSIL from HSIL is outdated and excessive.

The study by Valente and Schantz[9] identified some of the inequities in the grading scheme. The passing scores found in their workshop setting were comparable to the first administration of the Maryland Proficiency Test. [27-29] The differences in pass rates between technologists and pathologists seemed attributable to the grading scheme that allows partial or full credit to the technologist while penalizing the pathologist. Technologists receive full credit for identifying any abnormality (choices C or D) without being required to separate LSIL and HSIL (similar to CAP PAP and American Society for Clinical Pathology (ASCP) STAR scoring), while the pathologists lose half of the points allowed. While obvious LSIL and CIS/Cancer cases performed reasonably well in a workshop setting, some cases were close enough to the border between LSIL/CIN I and HSIL/CIN II to show poor separation of the C and D categories; the result was that some slides had about 60% correct answers while others had 80–90% consensus.

Based on the information above, we propose only a small penalty of one quarter of the points allowed when pathologists give an LSIL response for an HSIL case or vice versa.

Another area of obvious concern centers around the "A" choice- Unsatisfactory (Table 1). Only VERY obvious unsatisfactory cases elicited the desired response with only 60% of respondents in Valente and Schantz's [9] study getting the correct answer. In the CAP PAP program, less than 50% of the slides accepted into the program using the three Board certified anatomic pathologists' rule achieved field validation. [12] In addition, a considerable number of slides accepted as negative/NILM are reported as unsatisfactory by one or more participants. Based on TBS 2001 and ASCCP management guidelines, an unsatisfactory interpretation results in immediate repeat, and there is minimal detrimental effect to the patient if a negative "B" slide is reported as unsatisfactory. This consists chiefly of the inconvenience of having to return for a repeat test. Hence, there should be no penalty in the proficiency test if a negative slide is reported as unsatisfactory. However, the reverse situation, in which a field-validated unsatisfactory slide is reported as negative/NILM should carry a penalty, as such a patient would not receive early repeat.

Finally, we feel there is no justification that a false negative response of negative for an HSIL or cancer slide be given greater weight (minus 5) than a false positive response of HSIL for a negative slide (Table 1). Both pathologists and cytotechnologists should be given a score of zero when they give a negative response for an HSIL/cancer D category slide.

Based on the above reasoning and the published papers listed below, we propose the modification (Tables 3, 4) to the grading scheme to make it current with the triage algorithm and fair to the participants. Table 3, 4 show the proposed point values for a ten question test. Proportional changes should be made in the point values for a 20 question test.

**Table 3:** Point values (proposed)*

	**Pathologist (Technical Supervisor) 10 Slide Test**			
**Correct Response**	Examinee Response			

	A -UNSAT	B -NEGATIVE	C -LSIL	D-HSIL
A -Unsat	10	0	0	0
B -Negative	*10*	10	0	0
C -LSIL	5	0	10	*7.5*
D -HSIL+	0	*0*	*7.5*	10

**Table 4:** Point values (proposed)*

	**Cytotechnologist 10 Slide Test**			
**Correct Response**	Examinee Response			

	A -UNSAT	B -NEGATIVE	C -LSIL	D-HSIL
A -Unsat	10	0	5	5
B -Negative	*10*	10	5	5
C -LSIL	5	0	10	10
D -HSIL+	0	*0*	10	10

*CETC believes that stringently field validated slides are mandatory for a test to be fair and valid.

### New technologies

New technologies such as computer assisted and location guided screening have become available since the specifications of the test were initially published. In an increasing number of laboratories use of these technologies is routine, and screening of conventional Pap smears in the traditional manner is no longer performed. Testing of such laboratories in the manner described in the current regulations is totally inconsistent with the CLIA law stating:

"...swith such testing to take place, to the extent practicable, under normal working conditions."

The regulations and need to be revised to accommodate laboratories in which the use of these new technologies is "normal working conditions." Furthermore, the testing scheme should be designed in such a way that new technologies which come into use in the future, such as digital imaging, can be more readily accommodated. This should include technologies used in practice and in educational testing.

### Individual testing

One of the most troubling aspects of the statute is the requirement that cytotechnologists and pathologists be tested individually. While all other general proficiency testing under CLIA is directed toward measuring results at the laboratory level, this provision departs from that approach and singles out individuals. In many if not most laboratories, cytotechnologists and pathologists have the opportunity to consult their colleagues if they feel uncertain regarding the most appropriate interpretation of the slide. For this reason, CLIA's primary focus on laboratory proficiency testing is well placed. While we certainly recognize that the statutory language governing PT for gynecologic cytology mentions testing of individuals, it is equally important to note that language also specifies that the Secretary should establish quality assurance standards that "assure consistent performance by laboratories of valid and reliable cytological services...with such testing to take place, to the extent practicable, under normal working conditions." In our estimation, "normal working conditions" can be reflected in this examination only by allowing the collaborative team approach that is a fundamental aspect of the laboratory environment and most pathology practices. The regulation's premise that individuals conducting laboratory work are doing so in isolation and making determinations alone is false for most practitioners. Any PT program seeking to adequately assess true-to-life results must reflect this workplace reality in its testing approach. We believe that laboratory-level testing is both permitted under the law and is a better approach to ensuring quality laboratory results, and is more reflective of how Pap tests are performed in laboratories. The advantage of this approach is that the functioning of the laboratory quality assurance processes is also evaluated. Quality assurance procedures should allow any underperforming individuals to be detected by the laboratory. In fact, although the CLIA statute requires "periodic confirmation and evaluation of the proficiency of individuals involved in screening or interpreting cytological preparation..." it does not specify the manner in which this task is to be accomplished. This suggests that the proficiency of individuals need only be periodically confirmed and evaluated and that formal enrollment of the individuals in a proficiency testing program, in lieu of laboratory enrollment in such a program, would be unnecessary.

## Summary

The member organizations of the CETC feel strongly that there are significant flaws associated with the proposed proficiency test and its implementation. The most immediate modifications include lengthening the required testing interval, utilizing stringently validated and continuously monitored slides, changing the grading scheme, and changing the focus of the test from individuals to laboratory level testing, as described herein. Integration of new computer-assisted and location-guided screening technologies into the testing protocol is necessary for the testing program to be compliant with the current CLIA law. The regulation also needs to be flexible enough to accommodate new technologies that are implemented in laboratory practice, education, and administration of the test. The changes recommended in this document address the most immediate technical and scientific concerns with the current implementation of proficiency testing for gynecologic cytology. The CETC will be submitting a subsequent document following full review of the current regulations with recommendations for changes, justifications, and impact.

The following organizations endorse this document:

American Society of Cytopathology

International Academy of Cytology

American Society for Clinical Pathology

Papanicolaou Society of Cytology

American Society for Cytotechnology

The College of American Pathologists, respectfully, declines to endorse this document at this time but it supports the underlying criticisms of the existing regulatory framework of cytology PT program. The CAP believes that an alternative approach to the program must be developed to replace the existing program and that such an alternative will likely require statutory and regulatory modifications of CLIA.

## Competing interests

The author(s) declare that they have no competing interests.
